# Flurbiprofen axetil attenuates cerebral ischemia/reperfusion injury by reducing inflammation in a rat model of transient global cerebral ischemia/reperfusion

**DOI:** 10.1042/BSR20171562

**Published:** 2018-07-06

**Authors:** Huisheng Wu, Chaoliang Tang, Lydia Wai Tai, Weifeng Yao, Peipei Guo, Junmou Hong, Xin Yang, Xinyi Li, Zhao Jin, Jianjuan Ke, Yanlin Wang

**Affiliations:** 1 Department of Anesthesiology, Zhongnan Hospital of Wuhan University, Wuhan, China; 2 Department of Anesthesiology, Renmin Hospital of Wuhan University, Wuhan, China; 3 Department of Anesthesiology, The First Affiliated Hospital of University of Science and Technology of China, Hefei, China; 4 Department of Anesthesiology, The University of Hong Kong, Hong Kong, SAR China; 5 Department of Anesthesiology, The Third Affiliated Hospital of Sun Yat-sen University, Guangzhou, China; 6 Department of Cardiac Surgery, Renmin Hospital of Wuhan University, Wuhan, China

**Keywords:** brain edema, cerebral ischemia reperfusion injury, inflammation, Flurbiprofen axetil, stroke

## Abstract

Ischemic stroke has been ranked as the second cause of death in patients worldwide. Inflammation which is activated during cerebral ischemia/reperfusion (I/R) is an important mechanism leading to brain injury. The present study aimed to investigate the effect of flurbiprofen axetil on cerebral I/R injury and the role of inflammation in this process. Rats were subjected to sham operation or global cerebral I/R with or without flurbiprofen axetil (5 or 10 mg/kg). Global cerebral ischemia was achieved by occlusion of bilateral common carotid arteries combined with hypotension for 20 min followed by reperfusion for 72 h. Then the neurological deficit score, hippocampal cell apoptosis, levels of aquaporin (AQP) 4, AQP9, intercellular cell adhesion molecule-1 (ICAM-1), nuclear factor-κB (NF-κB), tumor necrosis factor (TNF-α), interleukin-1 β (IL-1β), thromboxane B2 (TXB2), and 6-keto-PGI1α were assessed. After reperfusion, neurological deficit score was significantly increased accompanied by severe neuronal damage (exacerbated morphological deficit, increased terminal deoxynucleotidyl transferase-mediated dUTP-biotin nick end labeling assay (TUNEL)-positive cells and cleaved caspase-3 protein expression in hippocampal CA1 region). Cerebral I/R injury also enhanced expressions of TNF-α, IL-1β, NF-κB, AQP4 and AQP9 as well as TXB2 and TXB2/6-keto-PGI1α. All these changes were reversed by pretreatment with flurbiprofen axetil. Flurbiprofen axetil protects the brain from cerebral I/R injury through reducing inflammation and brain edema.

## Introduction

Global cerebral ischemia, the most common clinical feature of circulatory arrest, leads to delayed neuronal damage in certain vulnerable brain areas, such as the hippocampal CA1 region in both patients and experimental animals [1,2]. Inflammatory responses (such as the release of proinflammatory cytokines interleukin-1 β (IL-1β), IL-6, and tumor necrosis factor (TNF-α)) increase the expression of adhesion molecules in white blood cells and vascular endothelial cells after brain ischemia and reperfusion [3]. These events following cerebral ischemia result in neutrophilic granulocyte migration and adhesion toward the endothelial cell of capillary, which cause tissue loss and neurological deficit in the brain [4].

Ischemia or hypoxia often induces brain edema that is classified as either cytotoxicity or vasogenicity. Aquaporin (AQP) is a water-transporting protein that serves as the major pathway for water transport across the plasma membranes in the brain [5]. AQP4 and AQP9 are expressed ubiquitously throughout the brain. Previous studies showed that AQP4 inhibition might provide a new therapeutic option for reducing brain edema. Together with AQP9, it also plays a critical role in the maintenance of water homeostasis, osmotic regulation, and energy metabolism in the brain as well as in brain edema after ischemic stroke [6,7].

Synthesis of thromboxane A2 (TXA2) increases while prostacyclin (PGI2) decreases after cerebral ischemia/reperfusion (I/R) injury. The augmented ratio of TXA2 to PGI2 will exacerbate aggregation of platelet and vasoconstriction that induces microcirculation disturbance that aggravates levels of secondary injury of cerebral ischemia [8,9].

Flurbiprofen axetil, one of the commonly used nonsteroidal anti-inflammatory drugs (NSAIDs), inhibits cyclooxygenase nonselectively. It is prescribed largely in the clinic due to its anti-inflammatory, analgesic, and antifebrile properties by inhibiting prostaglandin’s synthesis [10,11]. However, the role of flurbiprofen axetil in cerebral I/R injury is unknown. We, therefore, conducted the present study to explore the efficacy of flurbiprofen axetil on relief of inflammatory responses to global cerebral I/R injury in a rat model. Findings from the present study may provide new insights into the development of novel therapeutic strategies for cerebral I/R injury.

## Materials and methods

### Animals

Seventy-two adult male Sprague–Dawley rats (16–17 weeks) weighing 300–350 g were employed in the present study. Rats were fasted overnight with free access to water a day before the experiment. All experimental procedures were performed under the guidelines approved by the Institutional Animal Care and Use Committee of Wuhan University.

### Induction of global cerebral ischemia

By combining bilateral common carotid artery occlusion and arterial hypotension, global cerebral ischemia was established as described with slight modifications [12,13]. In brief, rats were anesthetized with chloral hydrate (350 mg/kg, i.p.) during surgical procedures. The right femoral artery was cannulated for physiologic monitoring, and the jugular vein was cannulated for infusion of drugs and blood withdrawal. Using a midline cervical incision, both common carotid arteries were isolated from adhering tissue and nerves, and silk threads (3 to 0) were placed loosely around them. Arterial blood pressure was monitored continuously, and blood gases and pH value were measured before and during ischemia. Mean arterial blood pressure (MAP) was lowered to 35–45 mmHg by blood withdrawal (2.1–2.4 ml/100 g body weight) through the jugular vein with a heparinized injector which was kept in 37.5°C water bath for later reinfusion. Both common carotid arteries were occluded by placing microaneurysm clips around the vessels for 20 min. Reperfusion was achieved by removing the clips and reinfusing the collected blood at a speed of 1.2 ml/min. Their rectal temperature was measured and maintained at 37.3–37.7°C during and 2 h after ischemia with a heat lamp. Rats were then returned to their cages and closely monitored until they recovered from anesthesia. Neurological function of rats were assessed with neurological deficit score 1 h before the experiment, and those with neurological deficit were excluded [14]. We chose this transient global ischemia model because it is a widely accepted procedure that affected most of the brain; thus, regional sampling problems associated with focal ischemia would not be a problem. In addition, this model would mimic some forms of human brain pathology such as those seen after cardiogenic shock.

### Grouping

Rats were randomly divided into four groups (*n*=18 per group): sham group, global cerebral I/R group, I/R + flurbiprofen axetil 5 mg/kg group (I/R + F5), and I/R + flurbiprofen axetil 10 mg/kg group (I/R + F10). Intravenous administration of flurbiproren axetil was performed at 15 min before inducing ischemia and the injection volume was in proportion to body weight of individual rat. For example, 10 mg/kg flurbiprofen axetil was intravenously injected in I/R + F10 group, while Sham and I/R group were intravenously injected with 1 ml/kg blank emulsion before subjecting to global cerebral ischemia. In I/R + F5 group, both 5 mg/kg flurbiprofen axetil and 0.5 ml/kg blank emulsion were intravenously injected before inducing ischemia.

Rats in the sham group were subjected to the same surgical procedures as I/R group without occluding the arteries. Twenty minutes after ischemia, rats were subjected to reperfusion and neurological deficit score was evaluated at 6, 24, and 72 h after reperfusion. After neurological deficit scoring at these three time points, rats were killed for tissue collection.

### Neurological deficit score

Neurological evaluation was performed at 6, 24, and 72 h after reperfusion by the same investigator who was not involved in the study. Consciousness, breathing, smell, vision and hearing, reflexes, motor function, overall activity, orientation, and presence of seizures were scored, according to the neurological deficit score system (0–100 scale; 0 = no deficit, 100 = most severe deficit) adapted from previous study [15,16]. Rats with neurological deficit were withdrawn before inducing ischemia. The chosen neurological deficit score system allows the identification of neurobehavioral deficits after cerebral ischemic injury, which is found to correlate well with other outcome end points, such as electrophysiologic recovery or histopathologic damage [17,18].

### Histopathological analysis in hippocampal CA1 at 24 h after global cerebral I/R

Brains of rats (*n*=6 rats per group) in all the four groups were removed immediately at 24 h after global cerebral I/R injury and were cut along the coronal plane at 4 mm behind chiasma. Then half of the forebrains were fixed by 4% paraformaldehyde and embedded in paraffin. Coronal sections (6-μm thickness) of the dorsal hippocampus (at the level of –3.8 mm from bregma) were stained with Hematoxylin and Eosin (HE).

### qPCR analysis

mRNA expressions of AQP4 and AQP9 in the discrete cortex, and mRNA expressions of intercellular cell adhesion molecule-1 (ICAM-1) and nuclear factor-κB (NF-κB) in the hippocampus were determined by qPCR. Total RNA was extracted from the discrete cortex or hippocampus (*n*=6 rats per group at each time point) using a Takara Genomic DNA Extraction Kit (TaKaRa Bio, Inc., Shiga, Japan) at 6, 24, and 72 h after reperfusion. Extracted total RNA was then reverse transcribed to generate cDNA. The reverse transcription reaction was then amplified using SYBR Green qPCR system (TaKaRa Bio Inc.). The fold change in relative mRNA expression was determined using the 2^−ΔΔ*C*^_t_ method and β-actin as an internal control [19]. All the forward and reverse primers used in the study were shown in Table 1.

**Table 1:** The forward and reverse primers used in the study

Genes	Primers	
	Forward	Reverse
*AQP4*	5′-TCCTTTGGCCCTGCAGTTATC-3′	5′-AGGCTTCCTTTAGGCGACGTT-3′
*AQP9*	5′-GGGTCCTATGATTGGTGCTTTC-3′	5′-CCCAGGATACTAACCACGAAAG-3′
*ICAM-1*	5′-GGTGGGCAAGAACCTCATCCT-3′	5′-CTGGCGGCTCAGTGTCTCATT-3′
*NF-κB*	5′-TCAATGGCTACACAGGACCA-3′	5′-CACTGTCAC CTGGAACCAGA-3′
β-actin	5′-CATCCGTAAAGACCTCTATGCCAAC-3′	5′-ATGGAGCCACCGATCCACA-3′

### RIA

To determine the serum concentrations of TNF-α and IL-1β in blood, a total amount of 1 ml blood was drawn from jugular veins in rats (*n*=6 rats per group at each time point) at 6, 24, and 72 h after reperfusion. Serum was obtained by centrifugation of collected blood sample at 4°C for 10 min at 3000 rpm. The serum samples were stored at −20°C before concentration analysis of TNF-α and IL-1β according to RIAy kit manual (Nanjing Jiancheng Bioengineering Institute, Nanjing, China).

To assess levels of thromboxane B2 (TXB2) and 6-keto-PGF1α in discrete cortex of rats (*n*=6 rats per group at each time point) at 6, 24, and 72 h after reperfusion, homogenized discrete cortex in cold dehydrated alcohol 0.1 ml and saline 0.9 ml was centrifuged at 4°C for 15 min at 3500 rpm. The resulting supernatants were processed according to instructions of RIA kits (Nanjing Jiancheng Bioengineering Institute, Nanjing, China) to determine the contents of TXB2 and 6-keto-PGF1α.

### Western blot analysis of caspase-3 protein activation in hippocampus

The equal amounts of protein extracted from isolated hippocampal fractions (*n*=6 rats per group at each time point) at 6, 24, and 72 h after reperfusion were resolved by 7.5–12.5% SDS/PAGE and transferred on to PVDF membrane for immunoblot analysis, as previously described [20].  Glyceraldehyde-3-phosphate dehydrogenase (GAPDH) was used as a loading control. The expression of caspase-3 was quantitated in triplicate per sample.

### Terminal deoxynucleotidyl transferase-mediated dUTP-biotin nick end labeling assay staining in hippocampus

The other half of the forebrains (*n*=6 rats per group) at 24 h after reperfusion was embedded in optimal cutting temperature compound (OCT) (Tissue-Tek), sectioned into 5-μm slides, and stained with a terminal deoxynucleotidyl transferase-mediated dUTP-biotin nick end labeling assay (TUNEL) staining kit (Abcam, U.S.A.). The hippocampal apoptotic cells were counted. Cell nuclei were stained with DAPI. All fluorescent images were examined using a Leica DM3000 microscope and recorded using a DFC 420 camera (Leica, Germany) [21].

### Statistical analysis

Statistical analyses were performed using SPSS software (SPSS 13.0, SPSS Inc, Chicago, IL). Data are expressed as mean ± S.E.M. using GraphPad 6 software (GraphPad Software, U.S.A.). Data were analyzed by one-way ANOVA followed by LSD multiple comparison tests as a post-hoc comparison. *P*-value <0.05 was considered significant.

## Results

### Physiological variables during I/R

As shown in Tables 2 and 3, no difference was observed in pH, PaO_2_, PaCO_2_, glucose concentration, and MAP amongst all groups before ischemia. Levels of pH, PaO_2_, PaCO_2_, glucose concentration in all groups remained unchanged after ischemia. In comparison with the sham group, level of MAP was significantly reduced in control, F5, and F10 groups during ischemia and 5 min after ischemia while it recovered to normal range at 10 min after ischemia.

**Table 2:** Physiologic variables of I/R subgroups

Characteristic	Treatment groups			
	Sham	I/R	I/R + F5	I/R + F10
Pre-ischemia				
PaO_2_ (mmHg)	107.4 ± 3.5	106.6 ± 3.8	108.1 ± 4.1	108.3 ± 4.3
PaCO_2_ (mmHg)	40.89 ± 2.4	40.39 ± 3.91	41.89 ± 3.18	39.78 ± 3.12
Hydrogen ion concentration	7.36 ± 0.04	7.35 ± 0.03	7.35 ± 0.03	7.36 ± 0.02
Glucose (mg/dl)	125.17 ± 3.40	125.67 ± 2.91	127.22 ± 5.87	125.83 ± 1.89
Post-ischemia				
PaO_2_ (mmHg)	107.8 ± 4.8	107.5 ± 3.5	109.6 ± 3.9	109.4 ± 3.8
PaCO_2_ (mmHg)	40.78 ± 2.39	40.11 ± 2.17	40.44 ± 2.28	40.61 ± 3.01
Hydrogen ion concentration	7.35 ± 0.02	7.35 ± 0.03	7.34 ± 0.03	7.35 ± 0.03
Glucose (mg/dl)	121.72 ± 5.13	122.67 ± 3.66	124.72 ± 6.49	124.44 ± 4.09

Values are mean (S.D.). All variables were similar between the groups.

**Table 3:** MAP (mmHg) variables of I/R subgroups

Groups	Pre-ischemia	Ischemia	5 min post-ischemia	10 min post-ischemia
Sham	107.4 ± 3.5	40.89 ± 2.4	7.36 ± 0.04	125.17 ± 3.40
I/R	106.6 ± 3.8	40.39 ± 3.91*	7.35 ± 0.03*	125.67 ± 2.91
I/R + F5	108.1 ± 4.1	41.89 ± 3.18*	7.35 ± 0.03*	127.22 ± 5.87
I/R + F10	108.3 ± 4.3	39.78 ± 3.12*	7.36 ± 0.02*	125.83 ± 1.89

Values are presented as mean ± S.E.M. *P<0.01 compared with sham group.

### Flurbiprofen axetil attenuates cerebral I/R-induced neurological deficit

**Figure 1:**
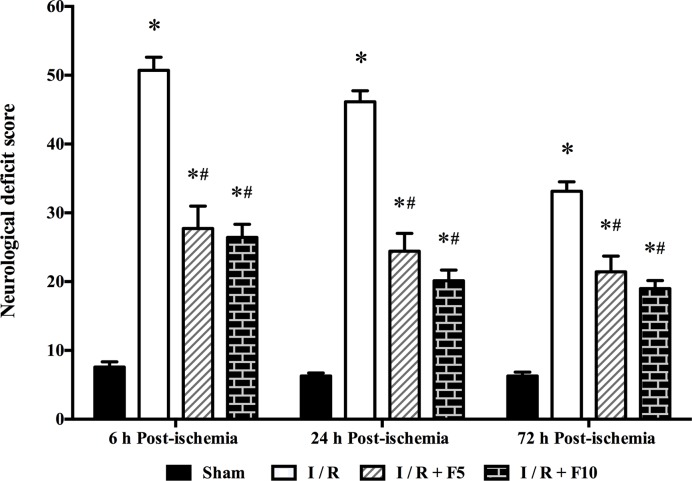
Neurological deficit score at 6, 24, and 72 h after reperfusion Sham group (Sham), global cerebral I/R model group (I/R), I/R + F5, and I/R + F10. Data were presented as mean ± S.E.M. *P<0.01 compared with sham group; ^#^*P*<0.01 compared with I/R group.

After cerebral I/R injury, rats exhibited significant neurological deficit manifested as elevated neurological deficit score at 6, 24, and 72 h after reperfusion compared with those in Sham group (*P*<0.01). Flurbiprofen axetil treatment at doses of 5 and 10 mg/kg significantly reduced neurological deficit score at 6, 24, and 72 h after reperfusion (*P*<0.01 compared with I/R); however, no difference was observed between F5 and F10 groups (Figure 1).

### Flurbiprofen axetil reduces cerebral I/R-induced neuronal damage

**Figure 2:**
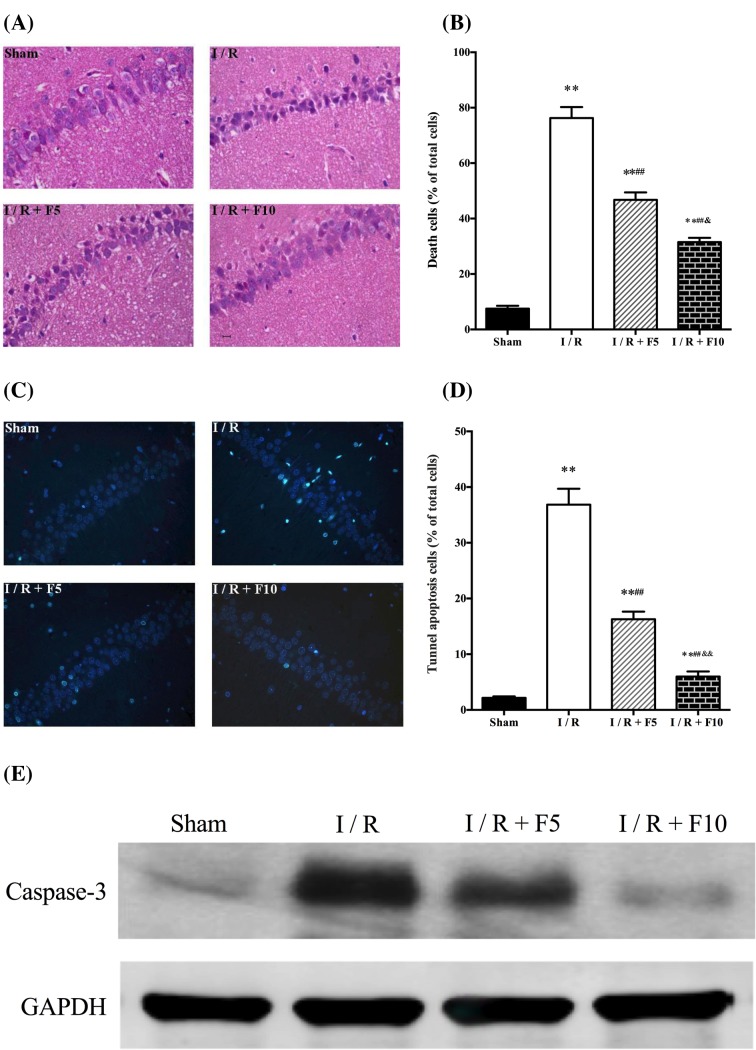
Flurbiprofen axetil reduced cerebral I/R-induced neuronal damage 24 h after global cerebral I/R (**A**,**B**) HE-stained sections of hippocampal CA1 regions (×200) and the percentage of necrotic cells. (**C**,**D**) TUNEL staining (×400) of hippocampal and number of positive cells; (**E**) caspase 3 protein expression measured by Western blot. Sham group (Sham), global cerebral I/R model group (I/R), I/R + F5, and I/R + F10. Data were presented as mean ± S.E.M. *P<0.05, **P<0.01, compared with sham group; ^#^*P*<0.05, ^##^*P*<0.01, compared with I/R group. ^&^*P*<0.01 compared with I/R + F5 group.

As shown in Figure 2, neuronal structures of hippocampal CA1 were normal in Sham group evidenced by a clear and tight order of three or four pyramidal cell layers with big and regular nuclei in the neurones. After cerebral I/R, hippocampal CA1 neurones were selectively and extensively damaged evidenced by pyramidal neuronal shrinkage and chromatin condensation of nuclei as well as reactive gliosis. This cerebral I/R-induced neuronal damage was reduced by flurbiprofen axetil treatment at both doses (*P*<0.01 compared with I/R) while treatment with flurbiprofen axetil at the dose of 10 mg/kg showed better effect than that of 5 mg/kg (*P*<0.01; Figure 2A,B). Moreover, cell apoptosis was significantly increased in hippocampal CA1 after cerebral I/R manifested as increased number of TUNEL positive cells and enhanced cleaved caspase-3 protein expression (*P*<0.01 compared with sham). These changes were attenuated by flurbiprofen axetil treatment, while treatment with flurbiprofen axetil at the dose of 10 mg/kg showed better anti-apoptotic effect than that of 5 mg/kg (*P*<0.01; Figure 2C–E, respectively).

### Flurbiprofen axetil reduces AQP4 and AQP9 in discrete cortex

**Figure 3:**
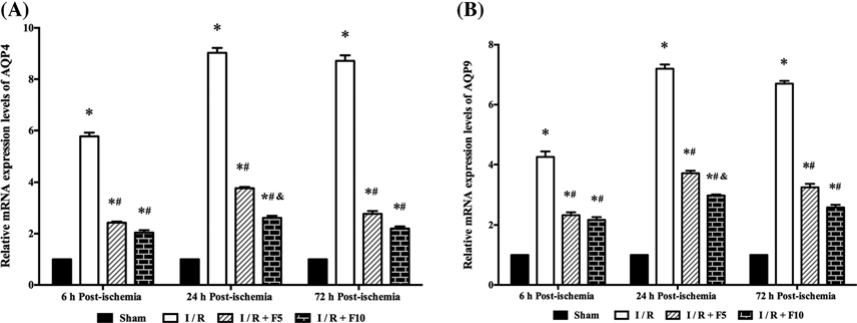
Flurbiprofen axetil reduced the mRNA expression levels of AQP4 and AQP9 Analysis of (**A**) *APQ4* and (**B**) *APQ9* mRNA expression levels by qPCR. β-actin was used as an internal control. Sham group (Sham), global cerebral I/R model group (I/R), I/R + F5, and I/R + F10. Quantitative data (*n*=3) are given as the mean ± S.E.M. *P<0.01 compared with Sham group; ^#^*P*<0.01 compared with I/R group; ^&^*P*<0. 01 compared with I/R + F5 group.

AQP proteins play a critical role in cerebral I/R-induced brain edema [22]. Two subtypes of AQP protein, AQP4 and AQP9 were detected by real-time PCR at 6, 24, and 72 h after reperfusion. As shown in Figure 3, *AQP4* and *AQP9* mRNA expression were significantly increased in discrete cortex 6, 24, and 72 h after reperfusion (*P*<0.01 compared with sham), which were reduced by pretreatment with flurbiprofen axetil (*P*<0.01). In addition, rats pretreated with 10 mg/kg flurbiprofen axetil had significantly lower *AQP4* and *AQP9* mRNA expressions than rats pretreated with 5 mg/kg flurbiprofen axetil at 24 h after reperfusion.

### Flurbiprofen axetil decreases cerebral I/R-induced inflammation in hippocampus

**Figure 4:**
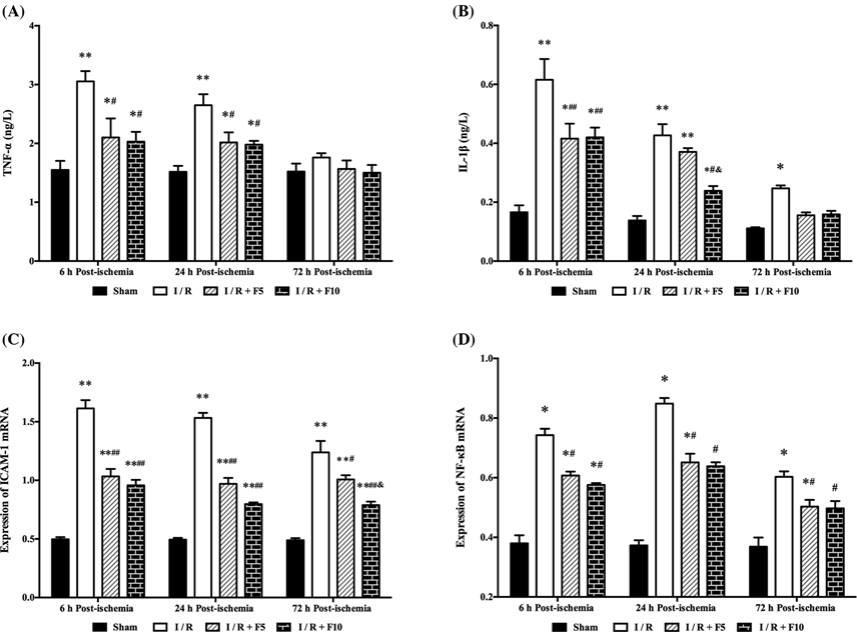
Flurbiprofen axetil reduced the expressions of proinflammatory factors Concentrations of (**A**) TNF-α and (**B**) IL-1β in serum, expression of (**C**) *ICAM-1* and (**D**) *NF-κB* mRNA in hippocampal at 6, 24, and 72 h after reperfusion. Sham group (Sham), global cerebral I/R model group (I/R), I/R + F5, and I/R + F10. Data were presented as mean ± S.E.M. *P<0.05, **P<0.01 compared with sham group; ^#^*P*<0.05, ^##^*P*<0.01 compared with I/R group; ^&^*P*<0.05 compared with I/R + F5 group.

Serum TNF-α concentration was significantly increased at 6 and 24 h after reperfusion (*P*<0.05 or 0.01, respectively.), which was significantly reduced by flurbiprofen axetil treatment (*P*<0.05, and *P*<0.05, respectively; Figure 4A). Serum IL-1β concentration was elevated at 6, 24, and 72 h after reperfusion and was reduced by pretreatment with flurbiprofen axetil at 6 and 24 h but not at 72 h after reperfusion (*P*<0.05 or 0.01, respectively.). In particular, the IL-1β level was significantly lower in 10 mg/kg flurbiprofen axetil-treated group than that in 5 mg/kg flurbiprofen axetil-treated group at 24 h after reperfusion (Figure 4B). *ICAM-1* and *NF-κB* mRNA expressions were significantly enhanced at 6, 24, and 72 h after reperfusion, which were reduced by pretreatment with flurbiprofen axetil (5 and 10 mg/kg) (*P*<0.05 or 0.01; Figure 4C,D, respectively).

### Flurbiprofen axetil reduces TXB2 and TXB2/6-keto-PGF1α in discrete cortex

**Figure 5:**
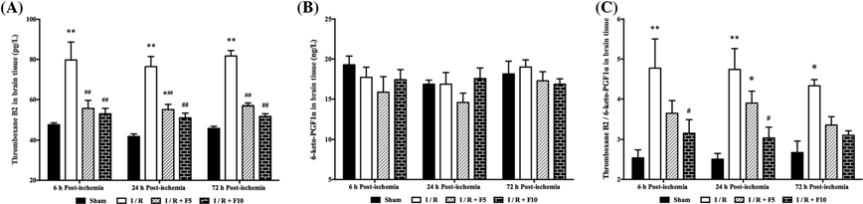
Concentrations of TXB2 and 6-keto-PGF1α in brain tissue Concentrations of (**A**) TXB2 and (**B**) 6-keto-PGF1α and (**C**) the ratio of TXB2 and 6-keto-PGF1α were measured at 6, 24, and 72 h after reperfusion. Sham group (Sham), global cerebral I/R model group (I/R), I/R + F5, and I/R + F10. Data were presented as mean ± S.E.M. *P<0.05, **P<0.01, compared with sham group; ^#^*P*<0.05, ^##^*P*<0.01, compared with I/R group.

As shown in Figure 5, level of TXB2 in discrete cortex was significantly increased at 6, 24, and 72 h after reperfusion (*P*<0.05 or 0.01 compared with sham, Figure 5A, respectively.), which were reduced by pretreatment with flurbiprofen axetil (5 and 10 mg/kg). However, no difference in 6-keto-PGF1α level amongst four groups was observed (*P*>0.05; Figure 5B). The ratio of TXB2/6-keto-PGF1α was significantly elevated at 6, 24, and 72 h after reperfusion (*P*<0.05 or 0.01 compared with sham, respectively) while rats pretreated with 10 mg/kg, but not 5 mg/kg flurbiprofen axetil, significantly reduced TXB2/6-keto-PGF1α ratio at 6 and 24 h after reperfusion (*P*<0.05; Figure 5C).

## Discussion

The present study successfully reproduced transient cerebral I/R injury model by inducing hypotension and occlusion of bilateral common carotid arteries in experimental SD rats. It is thought that persistent inflammation after I/R injury may cause ischemic neurone cell death contributing to the loss of neurological function observed in transient cerebral I/R injury [23]. In this study, as medications were given before induction of ischemia, the treatment was essentially preventive of the acute phase of transient cerebral I/R injury.

**Figure 6:**
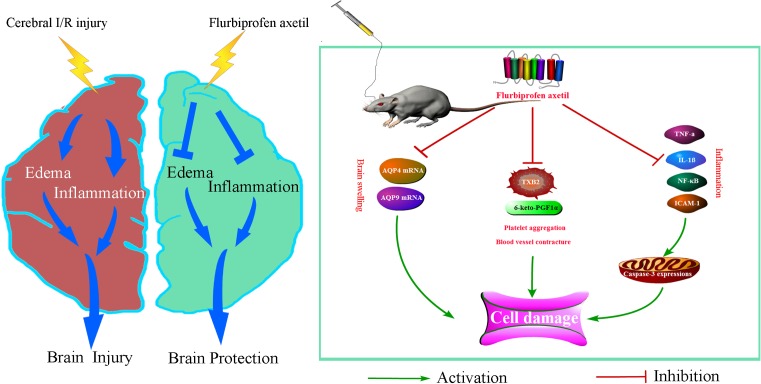
Proposed mechanisms for flurbiprofen axetil protects the brain from cerebral I/R injury through reducing inflammation and brain edema Cerebral I/R injury might facilitate the increase ininflammatory cytokines, *AQP* mRNA, and platelet aggregation and vessel contracture.

Flurbiprofen is a nonselective cyclooxygenase (COX) inhibitor used clinically as an NSAID [24]. NSAIDs such as flurbiprofen are prescribed largely for their anti-inflammatory, antipyretic, and analgesic properties. They function through inhibiting the activity of enzyme COX, which is related to prostaglandin synthesis. Flurbiprofen axetil is an injectable prodrug of flurbiprofen and is used mainly for postoperative pain control [25]. Clinical dosage of flurbiprofen axetil is 1–2 mg/kg while effective dose in rats is approximately 5–10 mg/kg [26,27]. The peak level of flurbiprofen axetil in blood reaches 5–10 min after intravenous administration, and exerts anti-inflammatory and analgesic effects at 15 min after intravenous administration. So intravenous injection of flurbiprofen axetil was conducted at 15 min before cerebral ischemic injury in the present study. The chosen model of global cerebral I/R injury in the present study would mimic some forms of human brain pathology such as those seen after shock, cardiac dysfunction, severe angiostegnosis, or angiemphraxis in the brain. Here, the results of neurological deficit score and histopathology at 6, 24, and 72 h after reperfusion indicated that the model of global cerebral I/R injury was successful. Moreover, neurological deficit scoring after reperfusion showed that flurbiprofen axetil pretreatment at 15 min before the injury improved neurological function in the cerebral I/R-injured rats, which indicated that flurbiprofen axetil might have protective effects on cognitive function.

As evidence-based practice has evolved, there appears to be at least two major recognizable pathways of ischemic cell death (i.e. necrotic cell death and apoptotic cell death) [28]. In a rat model of global cerebral I/R injury, the apoptotic neurones first occur in the hippocampal CA1 region that is the most sensitive region to ischemia in the brain. Previous studies had shown that the necrotic neurones appeared at 6–12 h after reperfusion with a peak at 24–72 h [29]. Thus, we picked time points at 6, 24, and 72 h after reperfusion to examine hippocampal CA1 region to determine the effect of flurbiprofen axetil on cerebral ischemia injury. Consistent with previous research where neurones of CA1 region were slightly damaged at 6 h after reperfusion [29,30]. Here, the neurones were extensively damaged at 24 and 72 h after reperfusion. However, some researchers had different views about the peak of apoptosis and necrosis, and the related mechanisms after cerebral I/R injury [31]. These differences could be explained by the differences amongst the models, the type and severity of the ischemia, species and age of the animals, and the observed region. The present study indicated that flurbiprofen axetil pretreatment can significantly reduce the number of dead neurones, and limit delayed neuronal death. Moreover, 10 mg/kg flurbiprofen axetil showed better protective effect than 5 mg/kg flurbiprofen axetil.

Brain edema caused by conditions such as trauma, ischemia, or hypoxia is often classified as either cytotoxic or vasogenic, resulting from an imbalance of water transportation in cells and blood vessels. Therefore, control of water transport and cell volume is of pivotal importance in modulating brain edema [32]. AQP4 is the most abundant and important water channel protein in the central nervous system (CNS) that expresses widely throughout the brain. AQP4 deletion has been found to protect adult mice brain from edema in stroke models, which suggests that AQP4 is closely related to edema. Another water channel protein, AQP9, is responsible for water homeostasis, osmotic regulation, and energy metabolism in the brain. It plays a critical role in brain edema after ischemic stroke and traumatic brain injury, and it is involved in the regulation of angiogenesis. In brain ischemia models, previous studies have shown that AQP9 could regulate post-ischemic edema [33]. Our study indicated that *AQP4* and *AQP9* mRNA expression were significantly increased in discrete cortex at 6, 24, and 72 h after reperfusion. However, they were reduced by pretreatment with flurbiprofen axetil. In addition, rats pretreated with 10 mg/kg flurbiprofen axetil had significantly lower *AQP4* and *AQP9* mRNA expression at 24 h after reperfusion than that pretreated with 5 mg/kg flurbiprofen axetil. The results may indicate that flurbiprofen axetil provides neuroprotection after global cerebral I/R injury by reducing brain edema in rats.

Cytokines are up-regulated in the brain after a variety of insults including global cerebral ischemia. They are expressed not only in cells of the immune system, but also production by resident brain cells, including glia and neurones [34,35]. The most important cytokines that are related to inflammation in cerebral ischemia are IL-1, TNF-α, IL-6, IL-10, and transforming growth factor-β (TGF-β) [36]. Amongst those cytokines, IL-1β and TNF-α appear to exacerbate cerebral injury. Previous report showed increased brain damage after IL-1β was administered to rats; moreover, mice with deficient in IL-1β had smaller infarcts than that in wild-type mice [37]. In addition, overexpression or treatment with IL-1 receptor antagonist reduces infarct size while IL-1 receptor deficient mice exhibit a dramatic increase in ischemic damage after I/R injury [38]. *IL-1β* mRNA elevations have been documented within 15–30 min after ischemia with increased IL-1β a few hours later [23]. Here, following 20-min transient global cerebral ischemia in rats, *IL-1β* mRNA and protein expressions were increased not only during early reperfusion (1 h), but also at later times (6–24 h) [39]. TNF-α is also up-regulated in the brain after ischemia with similar expression patterns as that of IL-1β. Inhibition of TNF-α reduces ischemic brain injury, while administration of recombinant TNF-α protein after stroke onset worsens ischemic brain damage [40,41]. Initial elevations are detected at 1–3 h after ischemia onset and, like IL-1β, have a biphasic pattern of expression with a second peak at 24–36 h [42,43]. Our data demonstrated that the concentrations of IL-1β (6, 24, and 72 h after reperfusion) and TNF-α (6 and 24 h after reperfusion) in serum were significantly enhanced after ischemia. However, they were suppressed by the flurbiprofen axetil pretreatment administered at 15 min before global cerebral ischemia, especially at dosage of 10 mg/kg. The results may indicate that flurbiprofen axetil provides neuroprotection after global cerebral I/R injury by inhibiting IL-1β and TNF-α in rats.

After cerebral ischemia, N-methyl-d-aspartate (NMDA) receptors are activated first in response to hyperstimulation [44]. This leads to a massive Ca^2+^ influx that activates the Ca^2+^-dependent phospholipases A2, which cleave membrane phospholipids to yield arachidonic acid (AA). AA is then converted by COXs into pro-inflammatory mediators such as the prostaglandins, TXB_2_, and the leukotrienes (LTs) that help to amplify the inflammatory response [45]. Of note, PGI2 and TXA2 are major metabolites of AA. PGI2, generated through the sequential activities of COX and prostacyclin synthase (PGIS), is a strong vasculoprotective prostanoid and is the predominant prostaglandin produced by endothelial cells [46]. TXA2 is the most labile prostanoid with a half-life of 30 s at 37°C, while the half-life of PGI2 is 4 min at 37°C. So they change into their metabolites (TXB_2_, 6-keto-PGF_1α_) swiftly. The level of TXB_2_ and 6-keto-PGF_1α_ are widely assayed to reflect the level of TXA2 and PGI2 in most studies [47]. It is important that the ratio of TXA2/PGI2 maintains a dynamic equilibrium in brain tissue for physiological functions. Production of oxygen radicals from I/R injury in brain tissue could injure vascular endothelial cell, inhibit activities of PGIS, and lead to vascular endothelial cell damage. Synthesis of TXA2 activation could lead to an imbalance of TXA2/PGI2 that induces capillary blockade by platelet, leukocyte, and sediments of fibrin. These events result in a phenomenon of ‘no reflow’ and microcirculation disturbance in the brain tissue, which may aggravate cerebral secondary injury [48].

Our study showed that the content of TXB2 in cortex increased significantly at 6, 24, and 72 h after global cerebral I/R injury, while the content of 6-keto-PGF_1α_ stayed stable at different time points. As a result, the ratio of TXB2/6-keto-PGF_1α_ increased significantly at different time points after reperfusion. Our results also showed that flurbiprofen axetil significantly reduced the post-ischemic production of TXB2 in the brain. In particular, flurbiprofen axetil at 10 mg/kg reduced TXB2 and the ratio of TXB2/6-keto-PGF_1α_ to a greater extent than that at 5 mg/kg at the three time points. As a result, flurbiprofen axetil may have an aspirin-like effect on TXB2 and TXB2/6-keto-PGF_1α_ ratio. It is also suggested to be a more prospective drug than aspirin in the treatment of cerebral ischemia [49]. Together, our findings indicate that flurbiprofen axetil protects the brain in part by decreasing TXB2 synthesis and promoting a favorable TXB2/6-keto-PGF_1α_ ratio that improves microcirculation and eliminates the phenomenon of ‘no reflow’.

There are several limitations in the present study. However, we did not use Doppler for cerebral blood flow detection due to the limitations of conditions. On the other hand, while flurbiprofen axetil was found to protect the brain from cerebral I/R injury by reducing inflammation and brain edema, the exact mechanism remains unclear. Also, the translational ability of our results remains to be further tested. Thus, future studies are needed to verify flurbiprofen axetil as a new strategy for neuroprotection in the clinic.

## Conclusion

In conclusion, flurbiprofen axetil preconditioning improves neurological function and neuronal survival after ischemia, which is associated with inhibition of IL-1β, TNF-α, and TXB2 synthesis, creating a favorable TXB2/6-keto-PGF_1α_ ratio in rats. This neuroprotective effect of flurbiprofen axetil suggests a potential clinical strategy for pre-ischemic conditioning, which could be beneficial for patients scheduled to undergo surgical procedures associated with an increased risk of perioperative brain ischemia (e.g. in cardiovascular surgery or neurosurgery). Our finding warrants further research to determine the clinical role for this new intervention for preemptive neuroprotection and to further clarify the molecular mechanisms involved.

## Abbreviations

AA: arachidonic acid
AQP: aquaporin
CNS: central nervous system
ICAM-1: intercellular cell adhesion molecule-1
IL-1β: interleukin-1 β
I/R: ischemia/reperfusion
I/R + F5: I/R + flurbiprofen axetil 5 mg/kg group
I/R + F10: I/R + flurbiprofen axetil 10 mg/kg group
MAP: mean arterial blood pressure
NF-κB: nuclear factor-κB
NSAID: nonsteroidal anti-inflammatory drug
PGIS: prostacyclin synthase
PGI2: prostacyclin
TNF-α: tumor necrosis factor
TUNEL: terminal deoxynucleotidyl transferase-mediated dUTP-biotin nick end labeling assay
TXA2: thromboxane A2
TXB2: thromboxane B2
